# Pilot PET study of vaginally administered bioadhesive nanoparticles in cynomolgus monkeys: Kinetics and safety evaluation

**DOI:** 10.1002/btm2.10661

**Published:** 2024-05-09

**Authors:** Molly K. Grun, Praveen Honhar, Yazhe Wang, Samantha Rossano, Minsoo Khang, Hee Won Suh, Krista Fowles, Harvey J. Kliman, Alessandra Cavaliere, Richard E. Carson, Bernadette Marquez‐Nostra, W. Mark Saltzman

**Affiliations:** ^1^ Department of Chemical & Environmental Engineering Yale University New Haven Connecticut USA; ^2^ Department of Biomedical Engineering Yale University New Haven Connecticut USA; ^3^ Department of Radiology & Biomedical Imaging Yale School of Medicine New Haven Connecticut USA; ^4^ Department of Obstetrics, Gynecology & Reproductive Sciences Yale School of Medicine New Haven Connecticut USA; ^5^ Department of Radiology, Division of Advanced Medical Imaging Research University of Alabama at Birmingham Birmingham Alabama USA; ^6^ Department of Cellular & Molecular Physiology Yale School of Medicine New Haven Connecticut USA; ^7^ Department of Dermatology Yale School of Medicine New Haven Connecticut USA

**Keywords:** biocompatible, biodegradable, microbicide, nanoparticle, vaginal drug delivery

## Abstract

Long‐lasting vaginal dosage forms could improve the therapeutic efficacy of vaginal microbicides, but achieving long‐term delivery to the vaginal canal has been a significant challenge. To advance understanding of vaginal dosage retention and biodistribution, we describe a method of noninvasive imaging with ^89^Zr‐labeled bioadhesive nanoparticles (BNPs) in non‐human primates. We additionally examined the safety of repeated BNP application. BNPs administered vaginally to cynomolgus monkeys were still detected after 24 h (1.7% retention) and 120 h (0.1% retention). BNPs did not translocate to the uterus or into systemic circulation. Analysis of inflammatory biomarkers in the vaginal fluid and plasma suggest that BNPs are safe and biocompatible, even after multiple doses. BNPs are a promising delivery vehicle for vaginally administered therapeutics. Further studies using the non‐human primate imaging materials and methods developed here could help advance clinical translation of BNPs and other long‐lasting vaginal dosage forms.

**Abbreviations**
BDL: below detection limit
BNP: bioadhesive nanoparticle
DFO: deferoxamine
HPG: hyperbranched polyglycerol
NHP: non‐human primate
NNP: non‐adhesive nanoparticle
OSEM: ordered subset expectation maximization
PEG: polyethylene glycol
PET: positron emission tomography
PLA: polylactic acid
PSF: point spread function
ROI: region of interest
TLC: thin layer chromatography
TOF: time‐of‐flight
Zr‐89: zirconium‐89

**Translational Impact Statement**

This work highlights the potential of bioadhesive nanoparticles as a safe and effective delivery vehicle for vaginally administered therapeutics. The use of non‐human primates in this pre‐clinical study provides important translational insights and represents an essential step towards advancing this technology towards clinical use. The findings of this study could pave the way for further research in this area and the development of long‐lasting vaginal dosage forms that could significantly improve clinical outcomes for patients.

## 1 INTRODUCTION

The female reproductive tract is the entry site for numerous viruses and bacteria that cause infections ranging from HIV to bacterial vaginosis.^1^ There is a long‐standing interest in developing topical therapeutic formulations to treat vaginal infections. Many dosage forms—such as creams, gels, tablets, films, and suppositories—have been developed; however, achieving long‐lasting delivery remains a challenge. For example, a study by Witter et al. compared the vaginal retention of nonoxynol‐9 in five commercial products. The products included two creams, a foam, a suppository, and a film. After 2 h, nonoxynol‐9 retention for the five products ranged from 7% to 19% of the initial dose, and after 12 h, the retention was only 1.5%–5.5%.^2^

To overcome these challenges, there is growing interest in the use of nanoparticles to protect small molecules and provide long‐term retention by penetrating through or adhering to the mucus barrier.^3^, ^4^ For example, it has been demonstrated that coating nanoparticles in a hydrophilic, neutrally‐charged polymer like polyethylene glycol (PEG) or poloxamers can significantly improve nanoparticle diffusion through mucus.^5^, ^6^, ^7^

We have recently described a bioadhesive nanoparticle (BNP) formulation that is retained in the vaginal canal for at least 72 h in mice, significantly outperforming the non‐adhesive, mucus‐penetrating analogue.^8^ The nanoparticles are formed with a block copolymer of polylactic acid (PLA) and hyperbranched polyglycerol (HPG). Under oxidative conditions, vicinal diols on the surface of the nanoparticles can be converted to aldehydes, which bond to protein amine groups through Schiff‐base interactions. We demonstrated in mice that these BNPs are non‐toxic and can be detected in vaginal epithelial cells and leukocytes for up to 72 h post administration. Because of these promising results, we were interested in testing this formulation in non‐human primates (NHPs), an animal model that is commonly used to assess the safety and efficacy of microbicides and other vaginally administered therapeutics for clinical translation.^9^, ^10^

Although nanoparticle‐based vaginal formulations have been extensively studied in mice,^4^, ^11^ there are very few studies on nanoparticle‐based dosage forms in NHPs—a critical animal model for pre‐clinical evaluation. Srinivasan et al. examined the vaginal safety and pharmacokinetics of the antiretroviral IQP‐0528 in pig‐tailed macaques.^12^ The drug was formulated in thin polymeric films with and without PLGA nanoparticles. Although films formulated with PLGA nanoparticles provided extended release in vitro, the in vivo concentration of IQP‐0528 in vaginal fluid was similar for both formulations over 24 h. Kish‐Catalone et al. evaluated liposomes (Novasomes 7474) as a delivery vehicle for ‐2 RANTES—an HIV inhibitor—in cynomolgus monkeys.^13^ Interestingly, the liposome alone provided a similar level of protection against simian/human immunodeficiency virus (SHIV) compared to the liposome containing ‐2 RANTES, suggesting a potential prophylactic effect of the carrier. Overall, there is a significant need for more research evaluating the retention and efficacy of nanoparticle‐based vaginal dosage forms in more clinically relevant animal models.

Here, we developed a novel method to study nanoparticle distribution after vaginal administration in cynomolgus monkeys (*Macaca fascicularis*). To track the distribution and retention of nanoparticles over time, we labeled BNPs with zirconium‐89 (Zr‐89)—a radioisotope with a half‐life of 3.3 days—to obtain [^89^Zr]Zr‐DFO‐BNP. We used positron emission tomography (PET) imaging to visualize the location and quantify the concentration of [^89^Zr]Zr‐DFO‐BNP within the body. Finally, we used histology and cytokine analysis to examine the safety of BNPs. This pilot study outlines a promising imaging model that can be used to improve our understanding of the kinetics and distribution of vaginal dosage forms. The results of this NHP study also demonstrate clinical potential of BNPs as a long‐lasting and safe delivery vehicle for topical application to the vagina.

## 2 RESULTS

### 2.1 Nanoparticle fabrication and characterization

PLA‐HPG non‐adhesive nanoparticles (NNPs) were prepared by a single emulsion method and converted to BNPs by oxidation of vicinal diols on the HPG surface. Deferoxamine (DFO) mesylate was subsequently conjugated to BNPs to facilitate chelation of Zr‐89 for PET imaging. The hydrodynamic diameter of NNPs, BNPs, and DFO‐BNP ranged from 98 to 143 nm (Table 1). The zeta potential of NNPs and BNPs was negative (−43 to −24 mV), but we observed a positive zeta potential (27 mV) after DFO conjugation.

**TABLE 1:** PLA‐HPG NNP, BNP, and DFO‐BNP characterization.

Nanoparticle	*n*	Size (nm)	PDI	Zeta potential (mV)
Blank NNP	2	114 ± 2.9	0.16 ± 0.00	−24.4 ± 3.4
FITC‐NNP	18	122 ± 2.3	0.17 ± 0.01	−28.9 ± 1.7
Blank BNP	3	97.8 ± 1.1	0.19 ± 0.02	−27.2 ± 4.9
FITC‐BNP	4	143 ± 8.9	0.19 ± 0.01	−43.4 ± 1.4
DFO‐BNP	3	97.6 ± 2.2	0.16 ± 0.02	27.1 ± 2.3

*Note*: Data is reported as mean ± standard deviation.

### 2.2 Characterization of [ ^89^Zr]Zr‐DFO‐BNPs

**FIGURE 1:**
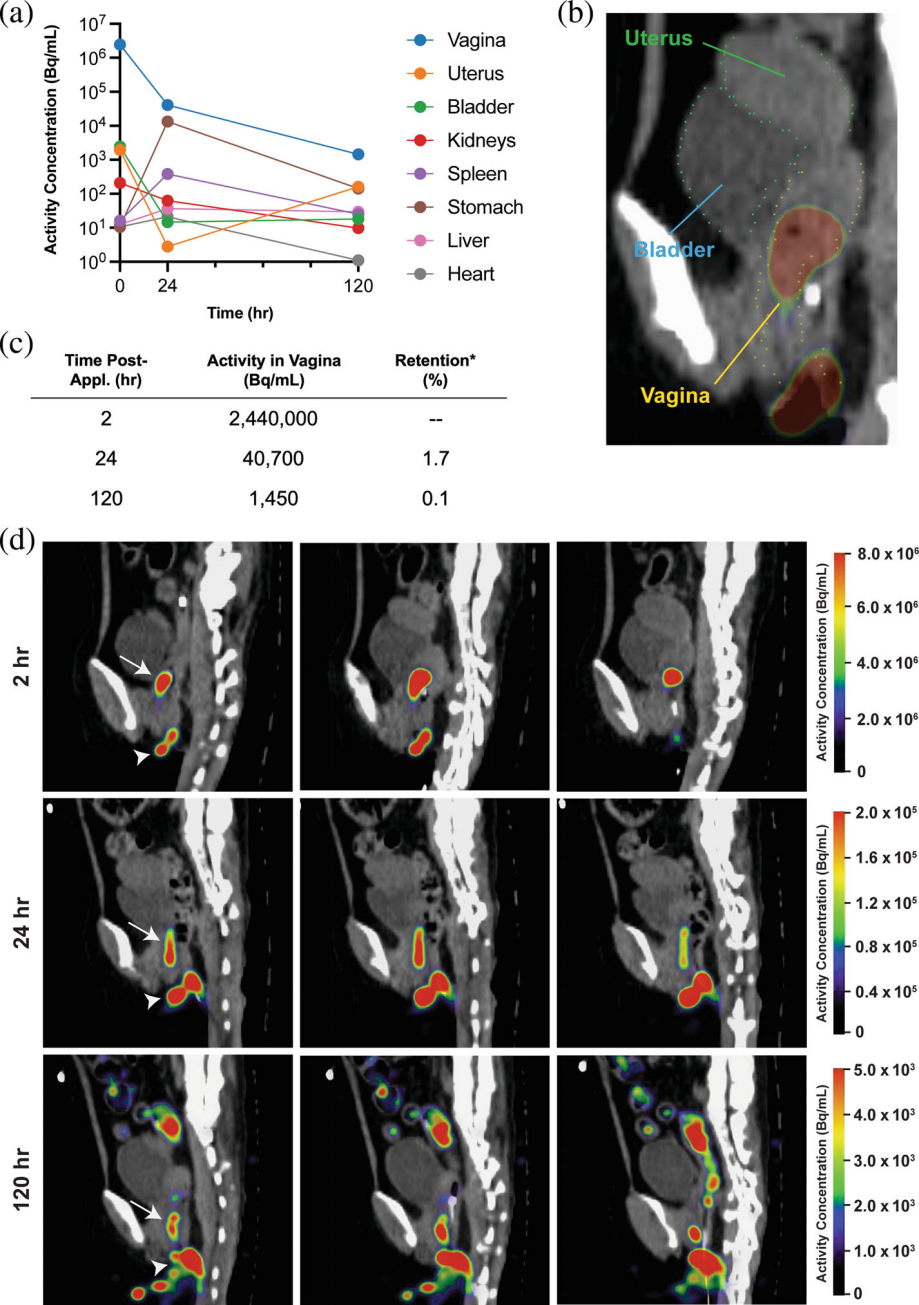
[^89^Zr]Zr‐DFO‐BNP retention and distribution after vaginal application (*n* = 1 with time points 2, 24, and 120 h after administration). (a) Organ‐specific activity for 120 h post vaginal administration. (b) Representative sagittal PET‐CT image of the reproductive tract with labeled organs. (c) Decay‐corrected activity in the vaginal canal over 5 days. *Retention is reported with respect to radioactivity concentration at 2 h and does not account for the  57% dose leakage during transport after dose administration (see description in Section 2.3: [^89^Zr]Zr‐DFO‐BNP retention and distribution). (d) Sagittal PET‐CT images of [^89^Zr]Zr‐DFO‐BNP in the vagina over 120 h. Arrows point to nanoparticles in upper vagina. Arrow heads point to leaked nanoparticles at vaginal opening.

The radiochemical yield of [^89^Zr]Zr‐DFO‐BNP was 99.3% as determined by radio‐TLC. [^89^Zr]Zr‐DFO was synthesized with a radiochemical yield of 99.6%. By in vitro testing, ~30% of [^89^Zr]Zr‐DFO released from BNPs over 6 days (Figure S1). However, our in vivo studies suggest the chelation is stable; [^89^Zr]Zr‐DFO‐BNP signal was retained in the vaginal canal, but we observed activity in multiple organs and the blood just 2 h after administration of the [^89^Zr]Zr‐DFO control (see below Figure 1 and Table 2).

**TABLE 2:** Decay‐corrected activity measured in blood samples during [^89^Zr]Zr‐DFO‐BNP and [^89^Zr]Zr‐DFO studies.

Radiotracer	Time post‐application (h)	Blood activity concentration (Bq/mL)
[^89^Zr]Zr‐DFO‐BNP	2	1.3
[^89^Zr]Zr‐DFO‐BNP	24	8.7
[^89^Zr]Zr‐DFO‐BNP	120	3.6
[^89^Zr]Zr‐DFO	2	990

### 2.3 [ ^89^Zr]Zr‐DFO‐BNP retention and biodistribution

**FIGURE 2:**
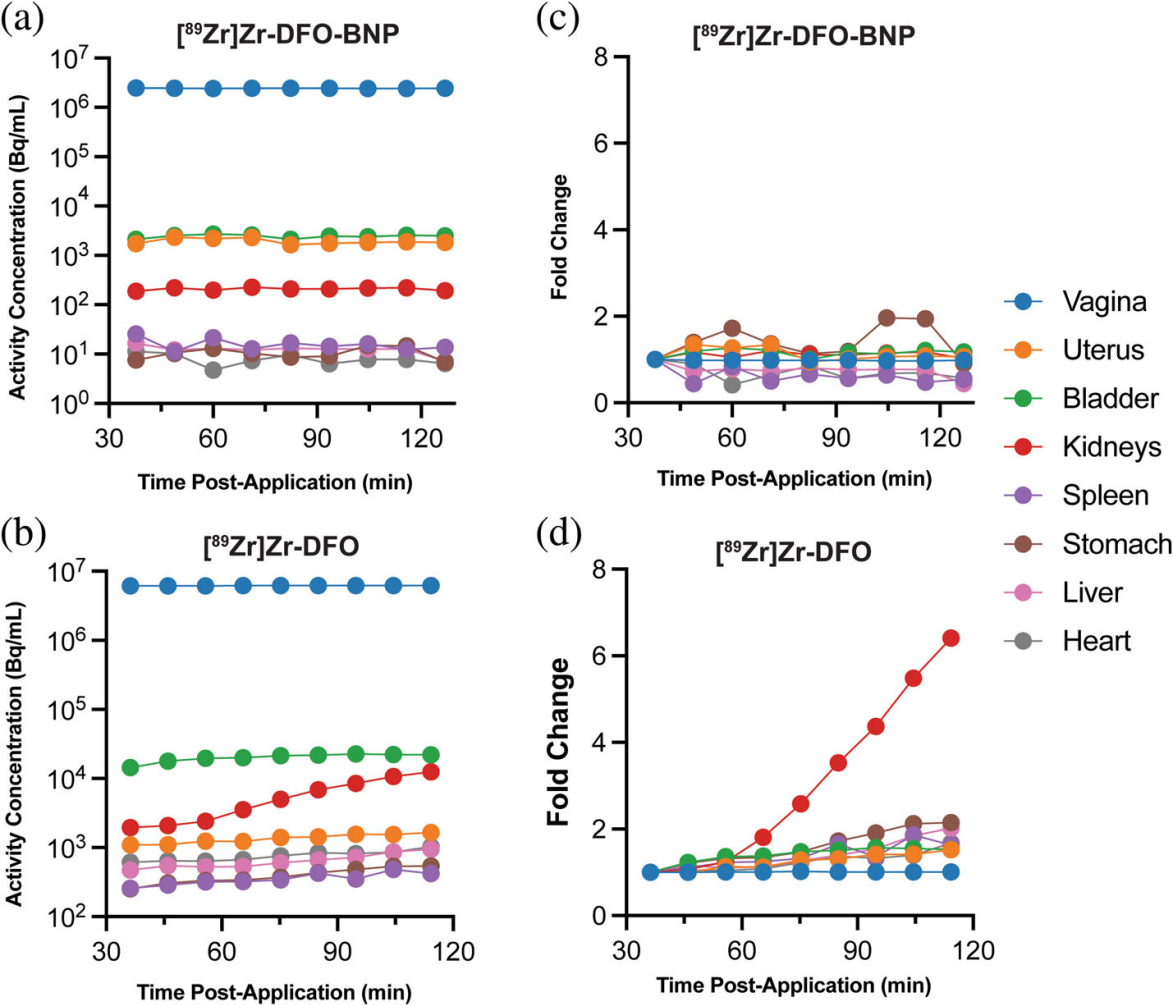
Kinetics of [^89^Zr]Zr‐DFO‐BNP and [^89^Zr]Zr‐DFO for 120 min after vaginal administration. Organ‐specific activity concentrations for (a) [^89^Zr]Zr‐DFO‐BNP (*n* = 1) and (b) [^89^Zr]Zr‐DFO (*n* = 1). Organ‐specific activity concentrations normalized to the first time point of each organ for (c) [^89^Zr]Zr‐DFO‐BNP (*n* = 1) and (d) [^89^Zr]Zr‐DFO (*n* = 1).

The stability of [^89^Zr]Zr‐DFO‐BNP in vivo was investigated by comparing its biodistribution with that of its potential fragment, the [^89^Zr]Zr‐DFO (650 Da) complex. To minimize partial volume effects in the bladder and uterus, regions of interest (ROI) in these regions were manually eroded to ensure separation from the vaginal ROI (Figure S2). For 120 min after vaginal application, [^89^Zr]Zr‐DFO‐BNP concentrations remained steady in the vagina and did not increase in most organs (Figure 2a,c). Notably, the activity in the kidneys remained steady at ~200 Bq/mL. In contrast, [^89^Zr]Zr‐DFO concentration in the kidneys increased starting around 60 min after vaginal administration (Figure 2b,d). By 120 min, kidney activity increased 6.4‐fold over the initial values (from ~2000 Bq/mL to 12,500 Bq/mL), suggesting that [^89^Zr]Zr‐DFO was taken up systemically from the vagina and filtered in the kidneys. The [^89^Zr]Zr‐DFO biodistribution observed here is consistent with prior work in mice.^14^

We further confirmed these findings by measuring radioactivity in blood samples taken directly after the 2 h scans. Blood activity levels in the [^89^Zr]Zr‐DFO‐BNP treated animal were negligible (1.3 Bq/mL at 2 h) compared to the [^89^Zr]Zr‐DFO treated animal (994 Bq/mL at 2 h, Table 2). Since kidney activity did not increase after [^89^Zr]Zr‐DFO‐BNP administration, these findings suggest that [^89^Zr]Zr‐DFO remains stably bound to the BNP after in vivo administration. They also indicate that BNPs did not translocate from the vagina into systemic circulation.

To quantify vaginal retention, [^89^Zr]Zr‐DFO‐BNP was administered topically to the vaginal canal, and whole‐body PET images were taken immediately after administration and after 24‐ and 120‐ h. Any [^89^Zr]Zr‐DFO‐BNP that leaked during administration and post‐procedure transport were collected in a diaper, which was measured with an additional PET scan to quantify the residual [^89^Zr]Zr‐DFO‐BNP. We estimate that 57% of [^89^Zr]Zr‐DFO‐BNP radioactivity was immediately lost to leakage during administration and post‐scan transport on day 0. The activity concentration in the vaginal canal after administration was 2.44 × 10^6^ Bq/mL, which decreased to 40,700 Bq/mL over 24 h—a retention of 1.7% (Figure 1). After 120 h, [^89^Zr]Zr‐DFO‐BNP was still detected in the vaginal canal at a concentration of 1450 Bq/mL, which is 0.1% of the administered dose.

**FIGURE 3:**
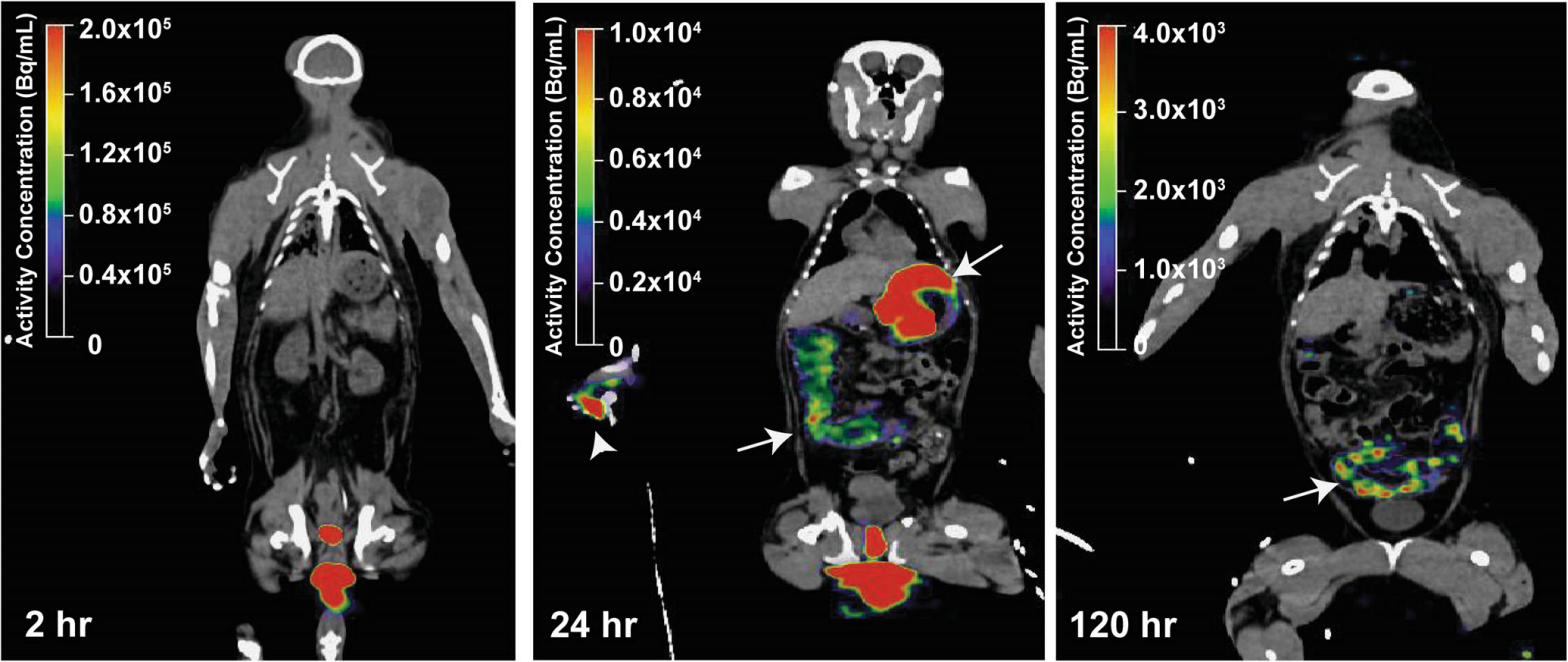
Off‐target [^89^Zr]Zr‐DFO‐BNP signal in the stomach and gastrointestinal tract at 24 and 120 h after administration. Arrows point to signal in stomach and intestines. Arrow head points to signal observed on hands. (*n* = 1 with time points 2, 24, and 120 h after administration).

[^89^Zr]Zr‐DFO‐BNP was primarily located in the upper vagina and around the surrounding skin of the vaginal opening (Figure 1d), likely due to leakage of excess dosage volume. On subsequent scans, we observed signal within the vagina and substantial activity on the external genitalia and surrounding skin. In addition to vaginal retention of [^89^Zr]Zr‐DFO‐BNP, we observed off‐target uptake in the stomach and digestive tract during the 24 and 120 h scans (Figure 3).

### 2.4 Vaginal safety

**FIGURE 4:**
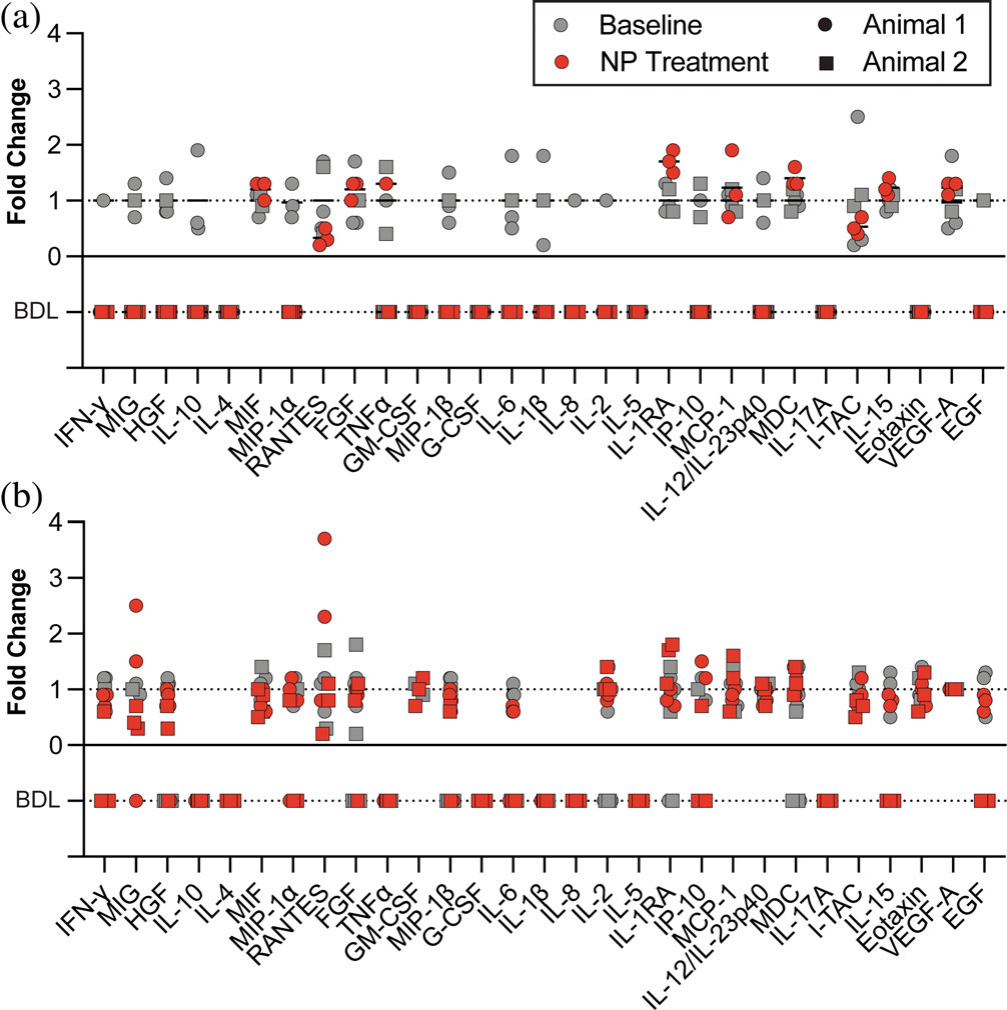
Changes in cytokine concentration in (a) vaginal fluid and (b) plasma after vaginal BNP administration. Samples that were below the detection limit (BDL) are identified under the x = 0 line. Fold change was calculated by normalizing all cytokine concentrations to the average baseline concentration for each animal. Values under the limit of detection were excluded. Two animals were used in the study. Baseline vaginal fluid and plasma samples were collected 33 days (Animal 1 only), 5 days, and directly prior to the first BNP administration. Nanoparticles were administered on day 0, day 2, day 4, and day 7. NP treatment samples were collected on day 2, day 4, and day 7 prior to the administration of BNPs on that day. A schematic of the delivery timeline can be found in Figure S3.

**FIGURE 5:**
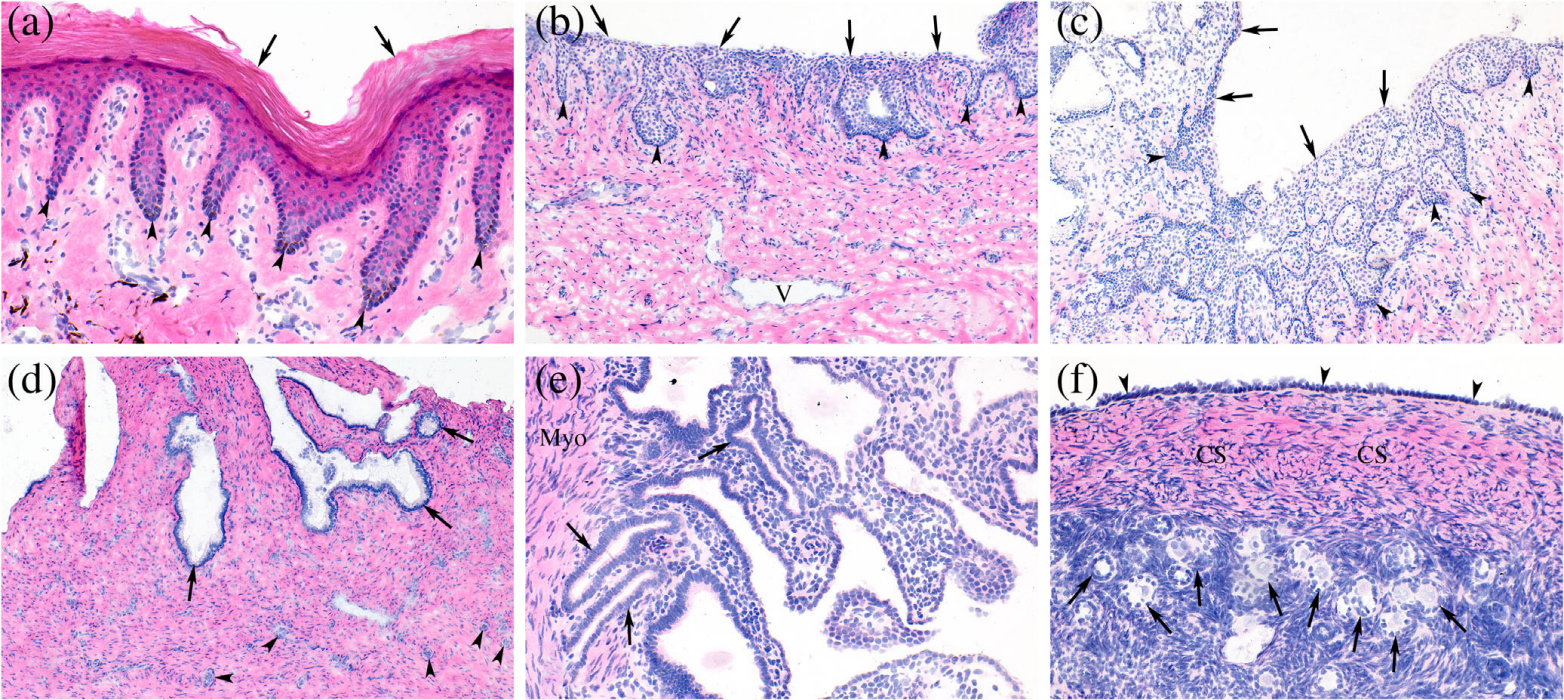
Histopathological survey of urogenital organs after intravaginal BNP administration (*n* = 2, samples taken after four BNP treatments over 7 days). (a) External genitalia (Animal 2) showing normal keratinized surface layer (arrows) and squamous papillae (arrow heads). (b) Vaginal surface (Animal 1) showing normal non‐keratinized surface (arrows) and squamous papillae (arrow heads). (c) Ectocervix (Animal 2) showing normal non‐keratinized surface (arrows) and squamous papillae (arrow heads). (d) Endocervix (Animal 1) showing normal endocervical glands (arrows) and small arterioles (arrow heads). (e) Uterus (Animal 2) at the myometrial endometrial junction revealing normal endometrial glands and adjacent myometrium (Myo). (f) Ovary (Animal 1) showing normal surface epithelium (arrow heads), cortical stroma (CS) and many normal appearing primary follicles, many of which contain oocytes (arrows). There was no evidence of neutrophilic or lymphocytic inflammation, erosions, fibrosis, or necrosis in any of the samples examined.

The safety of BNPs for vaginal use was evaluated by monitoring inflammatory biomarker levels in vaginal fluid and plasma before and after administration of multiple nanoparticle doses. There were no substantial differences in vaginal fluid cytokine levels, including important vaginal safety biomarkers such as TNFα, IL‐6, MIP‐1α, IL‐8, IL‐1RA, and IL‐1β (Figure 4a).^15^, ^16^ Several key cytokines, such as IFN‐γ and GM‐CSF, were below the detection limit for baseline and post‐treatment samples. To test for systemic reaction to BNP administration, we also monitored biomarker levels in plasma (Figure 4b). There were no substantial differences in expression for all cytokines tested. The raw data is available in the Supplementary Information (Table S1–S6). Urogenital tissue samples were collected at the end of the BNP delivery regimen and examined by a pathologist. No abnormal pathologic features were observed in any samples, and there was no evidence of neutrophilic or lymphocytic inflammation (Figure 5).

## 3 DISCUSSION

### 3.1 Evaluation of our findings

We produced and characterized BNPs to evaluate their biodistribution and residence time after vaginal administration to cynomolgus monkeys. To allow for imaging by PET, the BNP surface was modified by conjugation to a Zr‐chelator, DFO. Although DFO‐BNP has a different surface charge compared to unconjugated BNP, the positive zeta potential of DFO‐BNP might not reflect the surface charge of the administered particles ([^89^Zr]Zr‐DFO‐BNP). Unfortunately, we were not able to measure the surface potential of [^89^Zr]Zr‐DFO‐BNP since a dedicated zeta potential analyzer to measure radioactive samples was not available to us. If the surface charge of administered particles remains positive, the adhesion or uptake mechanisms could be affected by the difference in surface charge compared to unlabeled BNPs; however, the primary mechanism of BNP adhesion does not involve surface charge.^17^ The aldehyde groups on the surface of BNPs react with amines—such as the proteinaceous surface of the vaginal epithelium—to form Schiff‐bases. As long as the majority of aldehyde groups on the nanoparticle surface are not bound to DFO, they should be available for adhesion to proteins. Our previous work has the demonstrated that BNPs have an average of 17 aldehyde groups per polymer strand.^17^ Since the base polymer (PLA‐HPG) and DFO were conjugated at a 1:1 molar ratio, we estimate that 94% of aldehydes are free for bioadhesive reaction. Although the primary adhesion mechanism should not be affected by radiolabeling, further investigation could be considered to evaluate the impact of the inconsistencies in surface charge between radiolabeled and unlabeled BNPs on uptake and adhesion mechanisms.

Our in vivo studies suggest that ^89^Zr‐radiolabeling of BNPs is stable over our period of study. [^89^Zr]Zr‐DFO‐BNP was retained in the vaginal canal, whereas the [^89^Zr]Zr‐DFO control (a potential fragment [^89^Zr]Zr‐DFO‐BNP) was observed in multiple organs and blood samples 2 h after administration (Figure 2 and Table 2). This suggests that—since [^89^Zr]Zr‐DFO can be absorbed through the vaginal epithelium—[^89^Zr]Zr‐DFO remains bound to BNPs over the timescale of the experiment. One explanation for the discrepancy between in vitro and in vivo stability is that [^89^Zr]Zr‐DFO is released from BNPs so slowly under in vivo conditions that systemic uptake of released [^89^Zr]Zr‐DFO is too low to detect. It is also possible that the in vitro experimental design artificially increased release of [^89^Zr]Zr‐DFO through multiple wash and centrifugation steps.

We found that BNPs were retained for many days after a single vaginal dose. The observed vaginal retention of 1.7% at 24 h is comparable to commercial dosage forms, which—in one study—retained only 1.5%–5.5% of active pharmaceutical ingredient in the vaginal canal after 12 h.^2^ Although the fraction of BNPs retained after 5 days is low (~0.1% of administered volume), we believe even 0.1% BNP retention for this period could be therapeutically relevant. The 2018 FACTS‐001 Phase 3 clinical trial^18^ investigated the use of vaginally administered 1% tenofovir gel to protect against HIV‐1. Researchers estimated a threshold of protection equal to 30 ng/mL in 10 mL cervicovaginal lavage samples, which corresponds to roughly 300 ng retained tenofovir. Since the application dose was 40 mg tenofovir (4 mL of 1% tenofovir gel), the threshold of protection corresponds to a retention of approximately 0.00075%. Our observed BNP retention at 5 days (0.1%) exceeds this value. One limitation of this evaluation is that we are comparing retention of an active pharmaceutical ingredient to retention of the dosage form (nanoparticles). The rate of nanoparticle release could affect the retention of the encapsulant. However, since the retention of BNPs after 5 days is ~100‐fold larger than the retention of tenofovir at the estimated threshold of protection, we believe the observed BNP retention could have therapeutic potential. Despite these promising preliminary results, additional studies with a larger sample size are needed to make statistically significant conclusions about the performance of BNPs. These results will inform the design of future studies which could involve head‐to‐head comparisons with conventional drug dosage forms prior to clinical translation.

We noted that some signal from BNPs was observed on the external genitalia and surrounding skin. Retention on skin is consistent with our previous studies, which demonstrate that BNPs can effectively bind to the stratum corneum for several days.^17^ The binding of BNPs to the external genitalia is an important factor to consider for translation to human use. We also noted some signal in the stomach and digestive tract (Figure 3). We hypothesize that this uptake is from ingestion of [^89^Zr]Zr‐DFO‐BNP that leaked from the vaginal canal onto food that was consumed by the animal. This theory is further supported by the signal observed on the animal's hand during the 24‐h scan (see 24‐h panel in Figure 3). Apart from the vaginal canal, the next two regions with highest radioactivity during Day 0 scan were the bladder and uterus, though the signal was just 0.10% and 0.08% of the vaginal activity, respectively. This is likely attributed to partial volume effects (signal spill‐out from the vagina), rather than translocation of [^89^Zr]Zr‐DFO‐BNP, despite our efforts to minimize these effects by using smaller ROI masks for the bladder and uterus (Figure S2). Although translocation of fluid and sperm from the vagina to the uterus has been previously described in humans^19^ and cynomolgus monkeys,^20^ respectively, it is possible that translocation was not observed here because animals were induced into the luteal phase with depo‐provera injections. Antegrade contractions of the uterus during the luteal phase can prevent movement of fluid from the vagina to the uterus,^19^ which would likely confine particles to the vaginal canal. Further research is needed to understand the extent of BNP migration that could occur during different phases of the menstrual cycle.

We examined histological tissue specimens and measured fluid cytokine levels to evaluate the safety of this approach for vaginal drug delivery. Although we observed no substantial changes in cytokine levels after BNP treatment, it is important to note that many of the markers were below the limit of detection. In the case of vaginal fluid samples, this could be a result of the small sample volume (15–65 μL) and our subsequent dilution (5‐ to 20‐fold) into extraction buffer. An alternate approach is to collect cervicovaginal lavage samples and concentrate cytokines in filter tubes similar to Moss et al.^21^ In future studies, we suggest comparing analysis of both sample types (vaginal secretions and cervicovaginal lavage fluid) and performing further assay validation on vaginal fluid. Taken together, the results of the biomarker assessment and histopathological survey suggest that BNPs are non‐toxic and safe for vaginal use, which is consistent with our previous findings.^8^ However, the significance of these results is limited by the small sample size. In future studies, we also suggest including animals undergoing the same treatment without nanoparticles to rule out minor trauma resulting from sample administration and to use as a control for histopathological analysis.

### 3.2 Pilot study challenges and future suggestions

We encountered several challenges during the execution and analysis of this pilot study that could be improved in future work. First, the results and interpretation of this study are limited by the small number of PET imaging time points. Due to animal protocol requirements for recovery times from anesthesia, it was not possible to include an additional time point between 0 and 24 h. One possibility for obtaining data between 0 and 24 h is to sedate animals with short duration anesthesia for the vaginal application and then perform PET imaging at a later time such as 8 or 12 h. An additional time point between 24 and 120 h should be added to future studies. Second, we suggest administering a smaller volume of nanoparticles to improve the accuracy of retention calculations. We applied a volume of 0.5 mL, but a scan of the diaper used to contain dose leakage during the first scan suggests that 57% of the dose was lost immediately. The optimal dose volume would coat the vaginal epithelium without significant leakage. Alternatively, nanoparticles could be formulated in a gel or suppository to improve retention. We also suggest comparing BNP retention directly to conventional dosage forms, such as creams, gels, or suppositories. One major limitation of this study is the small sample size (n = 1) which does not allow for investigation of inter‐animal variation. Although the long‐lived radiotracer enabled longitudinal PET imaging—which is not possible during terminal studies that require several animals to obtain data at multiple timepoints—additional research and method optimization will be needed to fully characterize BNP safety and kinetics.

Imaging analysis could be improved by development of automatic ROI segmentation approaches for non‐human primate CT scans, which would ensure consistency and objectivity between scans. Finally, there was significant signal spill‐out from the vagina into the surrounding organs (uterus and bladder). To accurately quantify activity levels, we had to adjust ROI boundaries to exclude the region adjacent to the vaginal canal. Future studies could look at implementing partial volume correction algorithms to further minimize these effects.

## 4 MATERIALS AND METHODS

### 4.1 Nanoparticle preparation and BNP conversion

Non‐adhesive PLA‐HPG nanoparticles (NNPs) were prepared using a single emulsion, solvent evaporation process. 100 mg of PLA‐HPG polymer was dissolved overnight in 2.4 mL of ethyl acetate. The next morning, 0.6 mL of DMSO was added to the polymer solution, and the polymer solution was added dropwise to 4 mL of deionized water while vortexing (setting 10 on Vortex Genie 2, Scientific Industries). The emulsion was placed on ice and sonicated (3 × 10s with 10s on ice in between) using a probe sonicator. The solution was mixed with 10 mL deionized water and transferred to a round‐bottom flask, and organic solvent was removed using a rotary evaporator for 30 min. NNPs were then transferred to a centrifugal filter (Amicon Ultra‐15, 100 kDa MWCO, Sigma Aldrich) and centrifuged three times at 4000 × *g* to wash away excess polymer and solvent. After the final spin, nanoparticles were resuspended in deionized water and were flash frozen in liquid nitrogen and stored at −20°C until use. FITC‐labeled nanoparticles were prepared by substituting 10 mg (out of 100) of PLA‐HPG with FITC‐PLA (PolySciTech AV039). To prepare BNPs, NNPs were diluted to a concentration of 25 mg/mL, mixed with equal volumes of 10× PBS and 0.1 M NaIO_4_, and incubated on ice. After 20 min, one volume of 0.2 M Na_2_SO_3_ was added to quench the reaction. BNPs were then transferred to a centrifugal filter and washed three times at 4000 × *g*.

### 4.2 [ ^89^Zr]Zr‐DFO‐BNP and [ ^89^Zr]Zr‐DFO preparation

BNPs were labeled with Zr‐89 by conjugating a chelator, deferoxamine mesylate (CAS# 138–14‐7, Sigma), to the nanoparticle surface. Surface conjugation of DFO was achieved through reductive amination. After BNP conversion, BNPs (25 mg/mL) were incubated with 1 molar equivalent of DFO mesylate for 4 h at room temperature. Forty molar equivalents of NaCNBH_3_ were added, and BNPs were incubated for an additional 40 h. DFO‐BNP was washed four times to remove excess DFO mesylate and NaCNBH_3_ and diluted to 25 mg/mL. DFO‐BNP was then labeled with neutralized [^89^Zr]Zr‐oxalate in 0.25 M HEPES (pH 7.4) at a molar activity of 37 MBq/μmol and was incubated for 30 min at room temperature. Radiochemical yield was determined via radio‐thin layer chromatography (radio‐TLC), [^89^Zr]Zr‐DFO‐BNP was washed three times using a centrifugal filter (Amicon Ultra‐0.5, 100 kDa MWCO, Sigma Aldrich), and diluted to a final concentration of 222 MBq/mL (decay‐corrected to the time of delivery). To assess uptake without BNPs, [^89^Zr]Zr‐DFO was prepared as a control. 0.1 mg/mL DFO mesylate was labeled with neutralized [^89^Zr]Zr‐oxalate in 0.25 M HEPES (pH 7.4) at a molar activity of 2070 MBq/μmol and was incubated for 30 min at room temperature. Radiochemical yield was determined via radio‐TLC. [^89^Zr]Zr‐DFO was then loaded into an activated SEP‐PAK PLUS C18 cartridge (Waters Corp), washed twice with 1 mL deionized water, and eluted with 0.5 mL 95% ethanol. Excess ethanol was evaporated at 90°C for 1 h, and [^89^Zr]Zr‐DFO was diluted to a final concentration of approximately 222 MBq/mL (decay‐corrected to the time of delivery).

### 4.3 In vitro release

[^89^Zr]Zr‐DFO release from BNPs was assessed in vitro. After radiolabeling, [^89^Zr]Zr‐DFO‐BNP was diluted into simulated vaginal fluid^22^ at a pH of 5, 6, or 7 in order to cover the range of vaginal pH levels seen in humans and cynomolgus monkeys,^23^ and samples were then incubated at 37°C and 500 rpm. At pre‐determined time points (1, 2, 3, 6 days), [^89^Zr]Zr‐DFO was separated from BNPs with a centrifugal filter (Amicon Ultra‐0.5, 100 kDa MWCO, Sigma Aldrich). The filtrate activity was measured using a Hidex AMG automatic gamma counter (Hidex, Turku, Finland). Nanoparticles were re‐diluted in the corresponding simulated vaginal fluid and placed back on the incubator for the subsequent measurement.

### 4.4 NHP study plan and population

All NHP experiments were conducted under a protocol approved by the Yale University Institutional Animal Care and Use Committee (#11017). Two female cynomolgus monkeys (ages: 10, 12 years; weight: 5, 7.1 kg) were used in both studies. Prior to each PET scan, animals were sedated with alfaxalone (2 mg/kg), midazolam (0.3 mg/kg) and dexmedetomidine (0.01 mg/kg). Subsequently, anesthesia was maintained with 1.5%–2.5% isoflurane. Vital signs were monitored continuously. For the PET imaging study, [^89^Zr]Zr‐DFO‐BNP was administered to one animal and [^89^Zr]Zr‐DFO was administered to the other animal. For the toxicity study, both animals were treated with BNPs. Animals were given at least 4 weeks to recover between experiments.

Animals were injected with 30 mg of depo‐provera (medroxyprogesterone acetate) 25–30 days prior to the start of every study. Depo‐provera induces animals into the luteal phase of the menstrual cycle^24^ to minimize variability that might arise from differences in vaginal morphology. Additionally, the luteal phase epithelium in cynomolgus monkeys is most comparable to the human vaginal epithelium. During the follicular phase, the stratum corneum becomes keratinized. This extensive keratinization does not occur in the vaginal epithelium of humans during any phase of the menstrual cycle.^24^, ^25^ Inducing animals into the luteal phase—where the stratum corneum is not keratinized—provides the most translatable vaginal delivery comparison to humans.

### 4.5 PET imaging and quantification of radioactivity in blood samples

[^89^Zr]Zr‐DFO‐BNP (108 MBq, 3.5 mg, *n* = 1) or [^89^Zr]Zr‐DFO (100.6 MBq, 32.3 μg, *n* = 1) in a volume of 0.5 mL was administered vaginally on Day 0 to the monkeys using a 1 mL syringe prior to scanning on a Siemens Biograph mCT scanner. Animals were in supine position, and their pelvis was elevated to avoid leakage of tracer during scanning. For each PET scan, list‐mode data were acquired first in a stationary bed position over the animal's pelvis for 30 min, followed by 90 min of continuous‐bed‐motion whole‐body imaging, for a total of 120 min. For [^89^Zr]Zr‐DFO‐BNP, PET imaging was performed at 0, 24, and 120 h. For [^89^Zr]Zr‐DFO, PET images were acquired only on day 0, for 120 min after administration. PET images were reconstructed using time‐of‐flight (TOF) + point spread function (PSF) modeling with an ordered subset expectation maximization (OSEM) algorithm (2 iterations, 21 subsets). CT images were acquired in addition to PET scanning for attenuation and scatter correction, and for anatomical delineation. ROIs were manually defined on the CT image for the following regions: vagina, uterus, bladder, kidneys, spleen, stomach, liver, and heart. Time activity curves (30–120 min post‐injection) for day 0 are reported for both [^89^Zr]Zr‐DFO‐BNP and [^89^Zr]Zr‐DFO (units of Bq/mL) to examine their respective uptakes shortly after tracer administration. For longitudinal assessment of [^89^Zr]Zr‐DFO‐BNP kinetics, radioactivity concentration within each ROI was averaged throughout the PET scan for each day. Visage AI Accelerator (Visage Imaging, Inc., San Diego, CA) was used for ROI segmentation and figure generation. At the end of each scan, blood samples (6 mL from the saphenous vein) were collected in heparinized tubes for activity measurements using a Hidex AMG automatic gamma counter (Hidex, Turku, Finland).

### 4.6 Vaginal safety

To assess the safety of BNPs for vaginal application, 0.5 mL of FITC‐BNP (100 mg/mL in PBS) was applied topically to the vagina using a 1 mL syringe four times over the course of 1 week (days 0, 2, 4, and 7). Two animals were used in the study. Vaginal fluid and blood samples were collected 33 days (Animal 1 only) and 5 days before the start of the first delivery, and then immediately prior to nanoparticle application on each delivery day. A schematic of the delivery timeline and sampling plan can be found in the supplemental information (Figure S3). To collect vaginal fluid, pre‐weighed Weck‐Cel spears (Beaver Vistec International) were inserted partway into the vaginal canal and left in place for 5 min. The spears were then re‐weighed to calculate absorbed vaginal fluid (assuming a density of 1 g/mL) and stored at −80°C. On day 7, animals were euthanized 4 h after the final delivery, and collected tissue was fixed in 10% neutral buffered formalin for 7 days. Fixed samples were embedded in paraffin, cut into 5 μm sections, and stained with hematoxylin and eosin (H&E). Tissue sections were then examined by a pathologist for signs of abnormal features.

To prepare vaginal fluid samples for cytokine analysis, the swab tip was cut from the handle and placed in the top of a Spin‐X centrifugal filter (0.22 μm, Corning). 300 μL of elution buffer (PBS; 0.25% BSA; 1:100 dilution of protease inhibitor cocktail, Sigma P8340) was added, and the sample was incubated on ice for 30 min. Samples were then centrifuged for 20 min (16000 × *g*, 4°C), and the filtrate was collected and stored at −80°C. Blood samples were collected into EDTA‐coated collection tubes. Samples were centrifuged at 2000 × *g* for 5 min to collect plasma, which was stored at −80°C.

The cytokine content of vaginal fluid and plasma samples was analyzed with the Cytokine 29‐Plex Monkey Panel (Thermo Fisher Scientific) using a Luminex 200 instrument (Luminex Corporation). The dilution factor into extraction buffer [(300 μL buffer + volume of vaginal fluid collected)/(volume of vaginal fluid collected)] was used to calculate the concentration of each cytokine for vaginal fluid samples. Although ThermoFisher has not specifically validated Luminex assays for use with vaginal fluid, we followed a method of vaginal secretion preparation for multiplex cytokine assays described in previous publications.^26^, ^27^ Additionally, vaginal fluid samples were diluted 5‐ to 20‐fold into extraction buffer, likely minimizing any potential effect of biological sample interference. Due to the small number of animals and sample replicates—and the significant number of results below the detection limit—statistical analysis was not performed on the resulting data. Fold change was calculated by normalizing all cytokine concentrations to the average baseline concentration for each animal. Values under the limit of detection were excluded.

## 5 CONCLUSION

In this pilot study, we present a method of tracking vaginal retention of bioadhesive nanoparticles with excellent sensitivity over many days by conjugating a long‐lived radioisotope (Zr‐89, t_1/2_ = 3.3 day) to the particle surface. This method enables longitudinal tracking of nanoparticle retention and distribution throughout the reproductive tract without terminal studies that require several animals to obtain multiple timepoints. Using PET imaging, we found that BNPs were retained in the vaginal canal for at least 5 days and did not enter the uterus or systemic circulation. By contrast, a small molecule ([^89^Zr]Zr‐DFO) was detected in systemic circulation just 60 min after vaginal administration. Multiple vaginal doses of BNPs did not increase the expression of inflammatory biomarkers in vaginal fluid or plasma, suggesting that the nanoparticles are biocompatible. We note that the significance of these results is limited by the small sample size (*n* = 1 for kinetic study and *n* = 2 for safety study), therefore we cannot draw statistically significant conclusions about the kinetics and safety without additional experiments. In any case, these findings provide preliminary data from which future studies can be designed to determine optimal sample size, dose, and formulation to evaluate BNPs and other long‐lasting vaginal dosage forms.

## AUTHOR CONTRIBUTIONS

**Molly Grun:** Conceptualization; data curation; formal analysis; investigation; methodology; project administration; visualization; writing – original draft; writing – review and editing. **Praveen Honhar:** Data curation; formal analysis; writing – original draft; writing – review and editing. **Yazhe Wang:** Investigation. **Samantha Rossano:** Formal analysis; writing – original draft. **Minsoo Khang:** Investigation; methodology. **Hee Won Suh:** Methodology; resources. **Krista Fowles:** Project administration; resources; supervision. **Harvey Kliman:** Formal analysis; methodology; visualization. **Alessandra Cavaliere:** Investigation; methodology; writing – original draft; writing – review and editing. **Richard Carson:** Conceptualization; funding acquisition; resources; supervision; writing – original draft; writing – review and editing. **Bernadette Marquez‐Nostra:** Conceptualization; funding acquisition; methodology; project administration; resources; supervision; writing – original draft; writing – review and editing. **W. Mark Saltzman:** Conceptualization; funding acquisition; supervision; writing – original draft; writing – review and editing.

## FUNDING INFORMATION

This research was supported by a grant from the National Institutes of Health (R01 EB00487). PET imaging systems were acquired under NIH grant S10RR29245.

## CONFLICT OF INTEREST STATEMENT

YW, MK, HWS, and WMS are inventors on patent applications related to bioadhesive PLA‐HPG nanoparticles. WMS is a founder and consultant to Stradefy Biosciences, which is developing bioadhesive nanoparticles for other applications.

## Supporting information


**Data S1.** Supporting Information.

## Data Availability

The data that support the findings of this study are available on request from the corresponding author.
