# Supplementary material for: Pilot PET study of vaginally administered bioadhesive nanoparticles in cynomolgus monkeys: Kinetics and safety evaluation
Source: Bioeng Transl Med. 2024 May 9;9(5):e10661. doi: 10.1002/btm2.10661 (PMC11561825; doi:10.1002/btm2.10661)
Supplement: Supplementary file 1 — Data S1. Supporting Information. [file BTM2-9-e10661-s001.docx]

**Supplementary Information**

**Figure S1**: *In vitro* release of ^8^[^89^Zr]Zr-DFO from BNPs in simulated vaginal fluid at pH 5, 6, and 7. Error bars represent standard deviation of replicates (n=3).

**
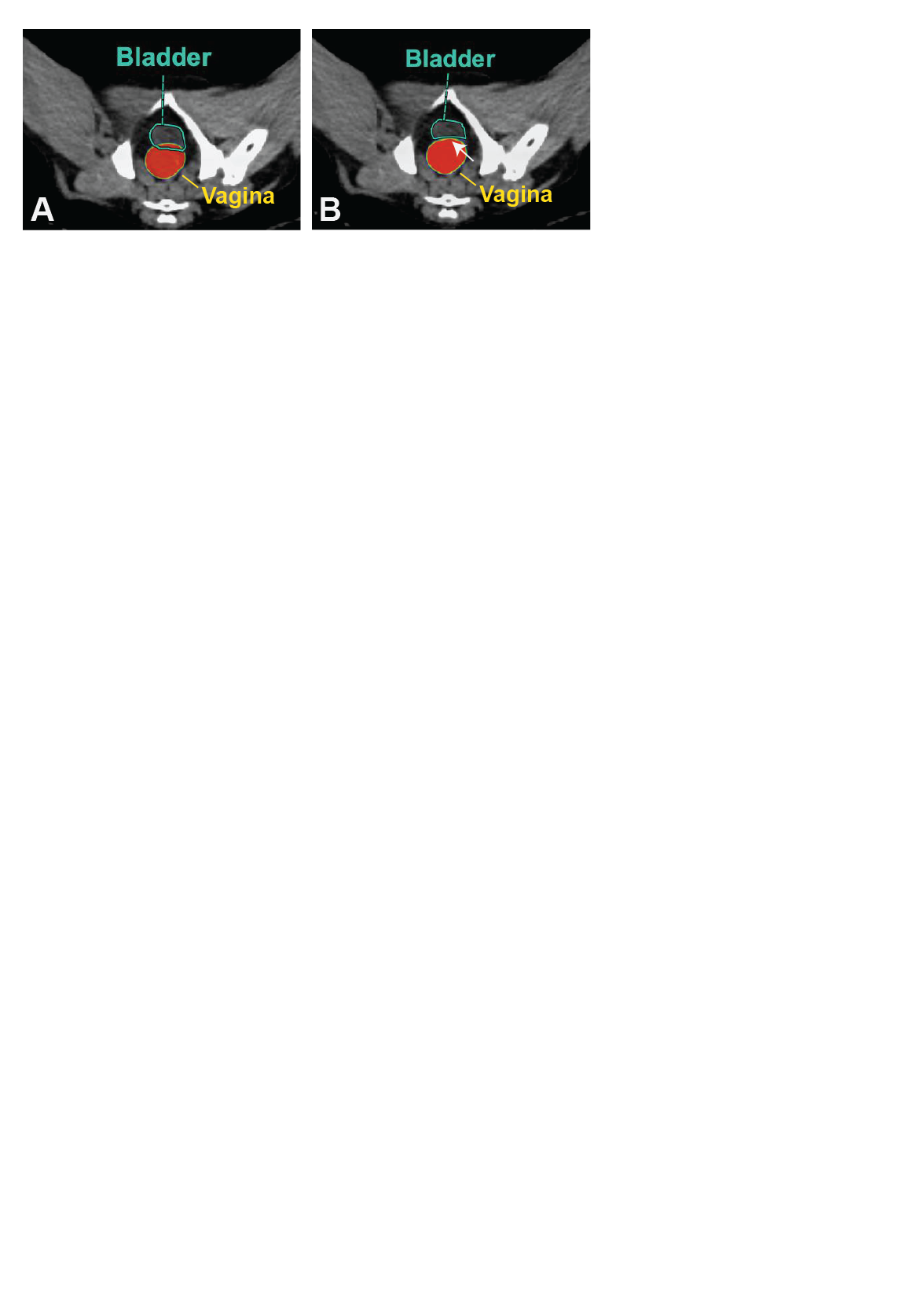
**

**Figure S2**: Example of manual ROI correction to minimize partial volume effect. **(A)** Original boundary for bladder. **(B)** Corrected bladder boundary. Arrow points to eroded region of bladder ROI.


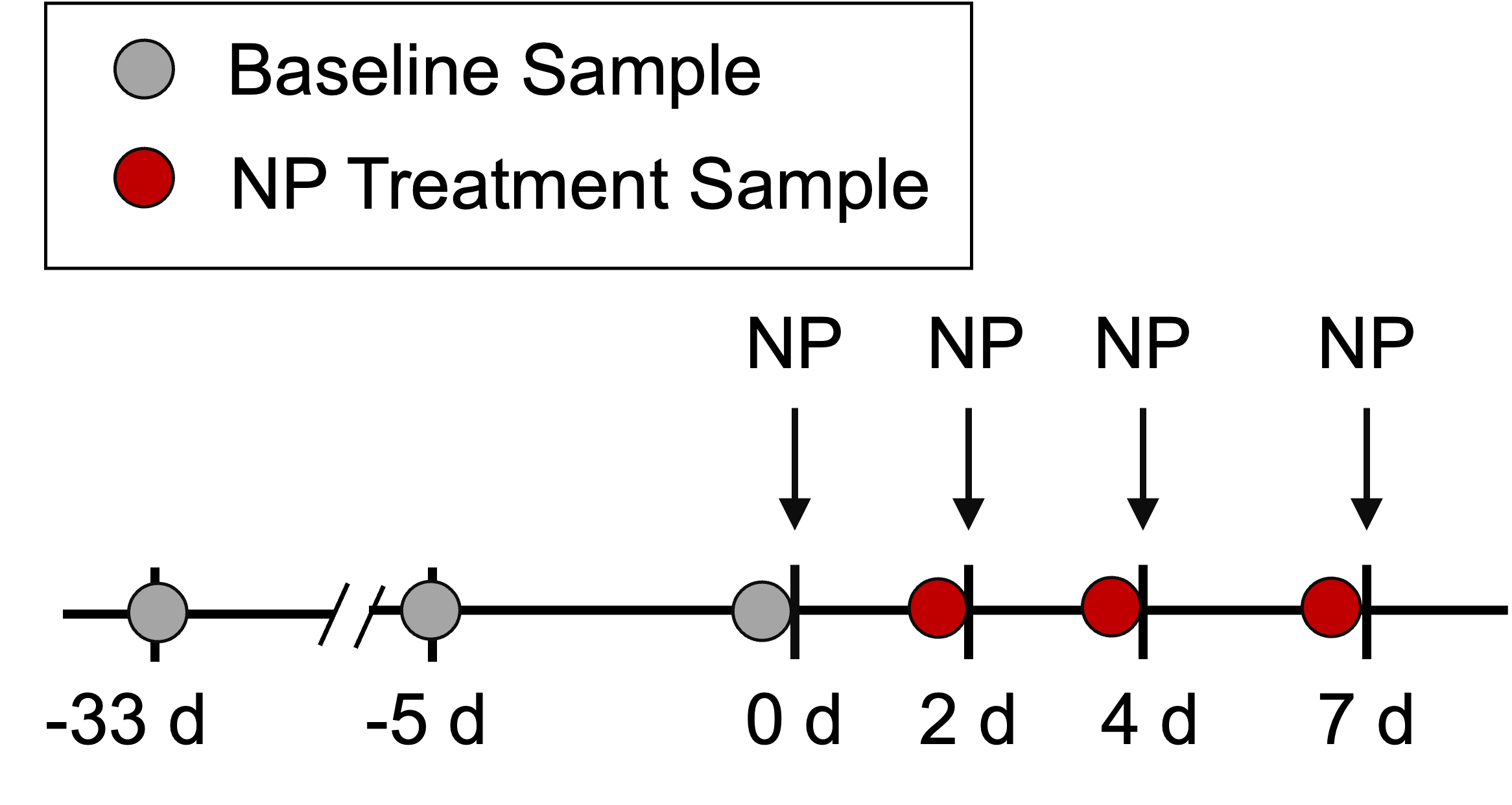


**Figure S3:** Vaginal safety study timeline. Two animals were used in the study. Baseline vaginal fluid and plasma samples were collected 33 days (one animal), 5 days, and directly prior to the first BNP administration. Nanoparticles were administered on day 0, day 2, day 4, and day 7. NP treatment samples were collected on day 2, day 4, and day 7 prior to the administration of BNPs on that day.

| **Cytokine** | **Baseline** | | | **After BNP Treatment** | | |
| --- | --- | --- | --- | --- | --- | --- |
|  | t = -33 d | t = -5 d | t = 0 d* | t = +2 d | t = +4 d | t = +7 d |
| IFN-g (pg/mL) | 12.21 | <10.36 | <10.36 | <10.36 | <10.36 | <10.36 |
| MIG (pg/mL) | 26.87 | 17.35 | <9.49 | 11.43 | <9.49 | 14.33 |
| HGF (pg/mL) | 143 | 96.03 | 53.97 | 35.67 | <28.03 | <28.03 |
| IL-10 (pg/mL) | 1.1 | 1.38 | 2.58 | 0.81 | <0.32 | <0.32 |
| IL-4 (pg/mL) | <8.69 | <8.69 | <8.69 | <8.69 | <8.69 | <8.69 |
| MIF (pg/mL) | 16132 | 10143 | 9424 | 11144 | 9940 | 5444 |
| MIP-1a (pg/mL) | 61.18 | 45.26 | 21.71 | 153 | <15.97 | 112 |
| RANTES (pg/mL) | 284 | 89.77 | 87.16 | 40 | 60.58 | 28.98 |
| FGF (pg/mL) | 132 | 53.23 | 30 | 30.47 | 65.64 | 13.64 |
| TNFa (pg/mL) | 12.78 | <7.39 | <7.39 | <7.39 | <7.39 | <7.39 |
| GM-CSF (pg/mL) | <4.45 | <4.45 | <4.45 | <4.45 | <4.45 | <4.45 |
| MIP-1b (pg/mL) | 172 | 118 | 46.38 | 324 | <8.43 | 381 |
| G-CSF (pg/mL) | <27.23 | <27.23 | <27.23 | <27.23 | <27.23 | <27.23 |
| IL-6 (pg/mL) | 15.87 | 44.91 | 8.13 | 58.13 | <3.58 | 104 |
| IL-1b (pg/mL) | 258 | <8.61 | 20.03 | 50.27 | <8.61 | 16.24 |
| IL-8 (pg/mL) | 10.07 | <5.54 | <5.54 | 11.03 | <5.54 | 8.98 |
| IL-2 (pg/mL) | 7.95 | <5.92 | <5.92 | <5.92 | <5.92 | <5.92 |
| IL-5 (pg/mL) | <4.05 | <4.05 | <4.05 | <4.05 | <4.05 | <4.05 |
| IL-1RA (pg/mL) | 85878 | 64237 | 36810 | 68138 | 37575 | 28973 |
| IP-10 (pg/mL) | 7.49 | <0.92 | <0.92 | <0.92 | <0.92 | 1.82 |
| MCP-1 (pg/mL) | 129 | 129 | 65.63 | 1147 | 27.67 | 1307 |
| IL-12/IL-23p40 (pg/mL) | 316 | 143 | 143 | <108.55 | <108.55 | <108.55 |
| MDC (pg/mL) | 300 | 285 | 179 | 359 | 179 | 919 |
| IL-17A (pg/mL) | <4.84 | <4.84 | <4.84 | <4.84 | <4.84 | <4.84 |
| I-TAC (pg/mL) | 2203 | 153 | 197 | 663 | 69.77 | 1397 |
| IL-15 (pg/mL) | 51.02 | 46.26 | 40.9 | 48.62 | 35.74 | 26.13 |
| Eotaxin (pg/mL) | <0.61 | <0.61 | <0.61 | <0.61 | <0.61 | <0.61 |
| VEGF-A (pg/mL) | 22.87 | 7.16 | 5.01 | 12.64 | 2.43 | 7.94 |
| EGF (pg/mL) | 81.43 | <8.63 | <8.63 | <8.63 | <8.63 | 202 |

* The sample at t = 0 d was collected just prior to nanoparticle treatment.

**Table S1:** Vaginal fluid cytokine expression for Animal 1.

| **Cytokine** | **Baseline** | | **After BNP Treatment** | | |
| --- | --- | --- | --- | --- | --- |
|  | t = -5 d | t = 0 d* | t = +2 d | t = +4 d | t = +7 d |
| IFN-g (pg/mL) | <10.36 | <10.36 | <10.36 | <10.36 | <10.36 |
| MIG (pg/mL) | 12.86 | <9.49 | <9.49 | <9.49 | <9.49 |
| HGF (pg/mL) | 53.97 | <28.03 | <28.03 | <28.03 | <28.03 |
| IL-10 (pg/mL) | <0.32 | <0.32 | <0.32 | <0.32 | <0.32 |
| IL-4 (pg/mL) | <8.69 | <8.69 | <8.69 | <8.69 | <8.69 |
| MIF (pg/mL) | 18062 | 7538 | 9727 | 9139 | 11692 |
| MIP-1a (pg/mL) | <15.97 | <15.97 | <15.97 | <15.97 | <15.97 |
| RANTES (pg/mL) | 215 | 25.5 | 20.8 | 18.28 | 37.61 |
| FGF (pg/mL) | 30.24 | 13.98 | 17.53 | 15.32 | 20.19 |
| TNFa (pg/mL) | 8.65 | 15.62 | 11.96 | <7.39 | <7.39 |
| GM-CSF (pg/mL) | <4.45 | <4.45 | <4.45 | <4.45 | <4.45 |
| MIP-1b (pg/mL) | 14.13 | <8.43 | <8.43 | <8.43 | <8.43 |
| G-CSF (pg/mL) | <27.23 | <27.23 | <27.23 | <27.23 | <27.23 |
| IL-6 (pg/mL) | 4.61 | <3.58 | <3.58 | <3.58 | <3.58 |
| IL-1b (pg/mL) | <8.61 | 8.95 | <8.61 | <8.61 | <8.61 |
| IL-8 (pg/mL) | <5.54 | <5.54 | <5.54 | <5.54 | <5.54 |
| IL-2 (pg/mL) | <5.92 | <5.92 | <5.92 | <5.92 | <5.92 |
| IL-5 (pg/mL) | <4.05 | <4.05 | <4.05 | <4.05 | <4.05 |
| IL-1RA (pg/mL) | 49857 | 41697 | 56657 | 54992 | 60911 |
| IP-10 (pg/mL) | 5.67 | 1.58 | <0.92 | <0.92 | <0.92 |
| MCP-1 (pg/mL) | 47.71 | 32.59 | 27.67 | 22.84 | 57.94 |
| IL-12/IL-23p40 (pg/mL) | 329 | <108.55 | <108.55 | <108.55 | <108.55 |
| MDC (pg/mL) | 194 | 134 | 164 | 164 | 164 |
| IL-17A (pg/mL) | <4.84 | <4.84 | <4.84 | <4.84 | <4.84 |
| I-TAC (pg/mL) | 786 | 449 | 278 | 181 | 250 |
| IL-15 (pg/mL) | 30.82 | 19.91 | 22.95 | 21.1 | 22.33 |
| Eotaxin (pg/mL) | <0.61 | <0.61 | <0.61 | <0.61 | <0.61 |
| VEGF-A (pg/mL) | 5.04 | 3.82 | 3.83 | 3.8 | 4.55 |
| EGF (pg/mL) | <8.63 | 11.03 | <8.63 | <8.63 | <8.63 |

* The sample at t = 0 d was collected just prior to nanoparticle treatment.

**Table S2:** Vaginal fluid cytokine expression for Animal 2.

| **Cytokine** | **Baseline** | | | **After BNP Treatment** | | |
| --- | --- | --- | --- | --- | --- | --- |
|  | t = -33 d | t = -5 d | t = 0 d* | t = +2 d | t = +4 d | t = +7 d |
| IFN-g (pg/mL) | 574 | 1085 | 1111 | 692 | 850 | 809 |
| MIG (pg/mL) | <9.49 | 14.33 | 12.86 | <9.49 | 20.46 | 33.48 |
| HGF (pg/mL) | 998 | 1745 | 1820 | 1091 | 1518 | 1427 |
| IL-10 (pg/mL) | <0.32 | <0.32 | <0.32 | <0.32 | <0.32 | <0.32 |
| IL-4 (pg/mL) | <8.69 | <8.69 | <8.69 | <8.69 | <8.69 | <8.69 |
| MIF (pg/mL) | 45.1 | 41.07 | 28.9 | 22.17 | 27.84 | 38.24 |
| MIP-1a (pg/mL) | 27.86 | 41.97 | 46.19 | 30.52 | 45.26 | 39.57 |
| RANTES (pg/mL) | 200 | 367 | 393 | 261 | 732 | 1187 |
| FGF (pg/mL) | 86.38 | 154 | 142 | 110 | 127 | 126 |
| TNFa (pg/mL) | <7.39 | <7.39 | <7.39 | <7.39 | <7.39 | <7.39 |
| GM-CSF (pg/mL) | <4.45 | <4.45 | <4.45 | <4.45 | <4.45 | <4.45 |
| MIP-1b (pg/mL) | 192 | 357 | 364 | 237 | 295 | 285 |
| G-CSF (pg/mL) | <27.23 | <27.23 | <27.23 | <27.23 | <27.23 | <27.23 |
| IL-6 (pg/mL) | 24.27 | 19.79 | 19.6 | 13.94 | 13.67 | 12.23 |
| IL-1b (pg/mL) | <8.61 | <8.61 | <8.61 | <8.61 | <8.61 | <8.61 |
| IL-8 (pg/mL) | <5.54 | <5.54 | <5.54 | <5.54 | <5.54 | <5.54 |
| IL-2 (pg/mL) | 133 | 181 | 76.84 | 144 | 99.73 | 111 |
| IL-5 (pg/mL) | <4.05 | <4.05 | <4.05 | <4.05 | <4.05 | <4.05 |
| IL-1RA (pg/mL) | 110 | 93.44 | 82.29 | 70.84 | 75.33 | 97.71 |
| IP-10 (pg/mL) | <0.92 | 1.32 | 2.06 | <0.92 | 2.06 | 2.53 |
| MCP-1 (pg/mL) | 798 | 1211 | 1187 | 820 | 1038 | 924 |
| IL-12/IL-23p40 (pg/mL) | 790 | 759 | 743 | 520 | 736 | 630 |
| MDC (pg/mL) | 2070 | 1310 | 1063 | 1275 | 1826 | 2139 |
| IL-17A (pg/mL) | <4.84 | <4.84 | <4.84 | <4.84 | <4.84 | <4.84 |
| I-TAC (pg/mL) | 41.48 | 49.99 | 54.31 | 35.28 | 45.71 | 59.79 |
| IL-15 (pg/mL) | 326 | 811 | 695 | 463 | 569 | 435 |
| Eotaxin (pg/mL) | 201 | 121 | 104 | 106 | 146 | 162 |
| VEGF-A (pg/mL) | <0.09 | <0.09 | <0.09 | <0.09 | <0.09 | <0.09 |
| EGF (pg/mL) | 30.05 | 86.16 | 82.21 | 42.93 | 57.4 | 52.81 |

* The sample at t = 0 d was collected just prior to nanoparticle treatment.

**Table S3:** Plasma cytokine expression for Animal 1.

| **Cytokine** | **Baseline** | | **After BNP Treatment** | | |
| --- | --- | --- | --- | --- | --- |
|  | t = -5 d | t = 0 d* | t = +2 d | t = +4 d | t = +7 d |
| IFN-g (pg/mL) | <10.36 | 19.94 | 12.21 | <10.36 | <10.36 |
| MIG (pg/mL) | <9.49 | 64.14 | 47 | 17.35 | 23.64 |
| HGF (pg/mL) | <28.03 | 539 | 361 | <28.03 | 156 |
| IL-10 (pg/mL) | <0.32 | <0.32 | <0.32 | <0.32 | <0.32 |
| IL-4 (pg/mL) | <8.69 | <8.69 | <8.69 | <8.69 | <8.69 |
| MIF (pg/mL) | 14.54 | 32.68 | 21.17 | 12.78 | 23.18 |
| MIP-1a (pg/mL) | <15.97 | 25.12 | 19.34 | <15.97 | <15.97 |
| RANTES (pg/mL) | 329 | 1830 | 1135 | 192 | 874 |
| FGF (pg/mL) | 2.07 | 20.48 | 12.94 | <0.5 | 9.52 |
| TNFa (pg/mL) | <7.39 | <7.39 | <7.39 | <7.39 | <7.39 |
| GM-CSF (pg/mL) | 19.09 | 15.37 | 21.24 | 11.76 | 17.22 |
| MIP-1b (pg/mL) | <8.43 | 13.78 | 10.33 | <8.43 | 8.95 |
| G-CSF (pg/mL) | <27.23 | <27.23 | <27.23 | <27.23 | <27.23 |
| IL-6 (pg/mL) | <3.58 | <3.58 | <3.58 | <3.58 | <3.58 |
| IL-1b (pg/mL) | <8.61 | <8.61 | <8.61 | <8.61 | <8.61 |
| IL-8 (pg/mL) | <5.54 | <5.54 | <5.54 | <5.54 | <5.54 |
| IL-2 (pg/mL) | 355 | 334 | 491 | 351 | 334 |
| IL-5 (pg/mL) | <4.05 | <4.05 | <4.05 | <4.05 | <4.05 |
| IL-1RA (pg/mL) | 18.49 | 42.6 | 50.95 | 32.47 | 54.35 |
| IP-10 (pg/mL) | <0.92 | 3.41 | 2.3 | <0.92 | <0.92 |
| MCP-1 (pg/mL) | 98.84 | 222 | 253 | 104 | 187 |
| IL-12/IL-23p40 (pg/mL) | 368 | 492 | 436 | 291 | 492 |
| MDC (pg/mL) | 285 | 697 | 677 | 447 | 560 |
| IL-17A (pg/mL) | <4.84 | <4.84 | <4.84 | <4.84 | <4.84 |
| I-TAC (pg/mL) | 45.71 | 92.42 | 56.5 | 37.33 | 45.71 |
| IL-15 (pg/mL) | <6.32 | <6.32 | <6.32 | <6.32 | <6.32 |
| Eotaxin (pg/mL) | 82.83 | 118 | 135 | 88.39 | 56.03 |
| VEGF-A (pg/mL) | <0.09 | <0.09 | <0.09 | <0.09 | <0.09 |
| EGF (pg/mL) | <8.63 | <8.63 | <8.63 | <8.63 | <8.63 |

* The sample at t = 0 d was collected just prior to nanoparticle treatment.

**Table S4:** Plasma cytokine expression for Animal 2.

| **Time Point** | **Dilution Factor** |
| --- | --- |
| -33 d | 12.6 |
| -5 d | 11.6 |
| 0 d | 19.5 |
| +2 d | 10.3 |
| +4 d | 13.3 |
| + 7 d | 5.6 |

**Table S5:** Vaginal fluid dilution factors for Animal 1.

| **Time Point** | **Dilution Factor** |
| --- | --- |
| -5 d | 9.0 |
| 0 d | 17.9 |
| +2 d | 19.9 |
| +4 d | 16.2 |
| + 7 d | 16.5 |

**Table S6:** Vaginal fluid dilution factors for Animal 2.
